# Blockade of D-serine signaling and adult hippocampal neurogenesis attenuates remote contextual fear memory following multiple memory retrievals in male mice

**DOI:** 10.3389/fnins.2022.1030702

**Published:** 2023-01-04

**Authors:** Ran Inoue, Xiance Ni, Hisashi Mori

**Affiliations:** ^1^Department of Molecular Neuroscience, Faculty of Medicine, University of Toyama, Toyama, Japan; ^2^Research Center for Idling Brain Science, University of Toyama, Toyama, Japan; ^3^Graduate School of Innovative Life Science, University of Toyama, Toyama, Japan

**Keywords:** D-serine, hippocampal neurogenesis, reconsolidation, multiple retrievals, remote memory

## Abstract

The retrieval of fear memories induces two opposing processes, reconsolidation, and extinction. The memory reconsolidation is an active process that involves gene expression and updates an existing memory. It is hypothesized that blockade of reconsolidation by manipulating the neurobiological factors, which are mechanistically involved in the process, could weaken or disrupt the original fear memory. The *N*-methyl-D-aspartate (NMDA) receptor and hippocampal neurogenesis play crucial roles in hippocampus-dependent memory processes, including reconsolidation. Using contextual fear conditioning paradigm with multiple retrievals, we attempted to weaken the original contextual fear memory by repeatedly disrupting retrieval-induced reconsolidation *via* downregulation of NMDA receptor signaling and inhibition of neurogenesis. In the first experiment, prior to fear conditioning, NMDA receptor signaling was downregulated by the genetic reduction of its co-agonist, D-serine, and the neurogenesis was dampened by focal X-ray irradiation on the hippocampus. We found that simultaneous D-serine reduction and neurogenesis dampening resulted in a progressive decrease in freezing following each retrieval, leading to an attenuation of remote contextual fear memory on day 28. In the second experiment using the same behavioral protocols, after conditioning, pharmacological approaches were conducted to simultaneously block D-serine signaling and neurogenesis, resulting in a similar suppressive effect on the remote fear memory. The present findings provide insights for understanding the role of D-serine-mediated NMDA receptor signaling and neurogenesis in memory retrieval and the maintenance of remote fear memory, and improving the efficacy of exposure-based therapy for the treatment of post-traumatic stress disorder (PTSD).

## Introduction

Pavlovian fear conditioning is a useful tool for studying the neurobiology of normal and pathological fear (Johnson et al., 2012; LeDoux, 2014; Bowers and Ressler, 2015). Contextual fear conditioning is a paradigm capable of creating fear memory to context and retrieving of the conditioned fear memory is hypothesized to share common or similar mechanisms with the re-experiencing symptoms in post-traumatic stress disorder (PTSD) (Bowers and Ressler, 2015; Chaaya et al., 2018; Schoenberg et al., 2022). Retrieval of the consolidated fear memory induces two opposing processes, reconsolidation and extinction. Brief retrieval of the fear memory (e.g., by less than 5 min re-exposure to the context) induces reconsolidation, whereas extended retrieval (30 min re-exposure) causes an extinguishing effect in mice (Suzuki et al., 2004). Reconsolidation maintains or enhances, while extinction weakens a retrieved fear memory (Suzuki et al., 2004). Studies have demonstrated that memory retrieval by re-exposure to context can return the memory to a temporary labile state, requiring an active restabilization process in order to persist (Nader et al., 2000). Reconsolidation is an active process that involves gene expression and updates an existing memory such as modifying memory strength, adding new information to an original memory (Nader and Einarsson, 2010). Therefore, it is hypothesized that reconsolidation prevention or blockade, by manipulating relevant neurobiological factors mechanistically involved in the process at either the molecular or cellular level, could weaken or even disrupt original fear memories (Careaga et al., 2016; Kida, 2019).

The NMDA receptors play essential role in synaptic plasticity, synapse formation, and neuronal excitation, through controlling Ca^2+^ ion flow into the cells (Cull-Candy et al., 2001). The receptor is composed of heterotetrameric subunit assemblies containing two GluN1 subunits that bind glycine or D-serine, and two GluN2 subunits that bind glutamate (Karakas and Furukawa, 2014; Lee et al., 2014). GluN2A and GluN2B are the most common NMDA receptor subtypes, localized in the cortical and hippocampal regions of the adult brain, and were identified as crucial synaptic elements in long-term memory formation, including the associative learning of fearful events (Monyer et al., 1994; Yuan et al., 2009). GluN2A-containing NMDA receptors are reportedly composed of 70–75% of the NMDA receptor population at CA3-CA1 synapses in the adult rat (Matsui et al., 1995), and silencing these synaptic NMDA receptors offers neuroprotection against NMDA receptor-induced excitotoxicity (Papouin et al., 2012). The small proportion of GluN2B-containing NMDA receptors at the postsynaptic density might be a necessary condition for the strengthening of individual synapses, and evidence suggests that the GluN2B subunit might be particularly important for plasticity and may make a synapse bidirectionally flexible, in serving to the induction of long-term potentiation (LTP) or long-term depression (LTD) (Matsui et al., 1995). In regard to the role of NMDA receptor subtypes in contextual fear conditioning, GluN2B-containing NMDA receptors are indispensable for memory reconsolidation and disrupting GluN2B-containing NMDA receptors could arrest destabilization induction (Milton et al., 2013). GluN2A-containing NMDA receptors in the retrosplenial cortex are required for the retrieval of recent and remote fear memory (Corcoran et al., 2011). The activation of a series of key molecules in the NMDA receptor signaling pathway in postsynaptic neurons, including cyclic adenosine monophosphate (cAMP) responsive element binding protein (CREB), is required for memory reconsolidation (Kida et al., 2002; Wang and Peng, 2016). Therefore, NMDA receptor signaling downregulation or blocking is an alternative to inhibit fear memory reconsolidation.

The GluN1 subunit is an obligatory subunit in all functional NMDA receptors and is thus widely expressed in all central neurons (Hansen et al., 2018). The synaptic receptors at CA3-CA1 areas, gated by glutamate and D-serine, are essential for LTP induction, which could be inhibited by degradation of extracellular D-serine *via* treatment of D-amino acid oxidase in slices (Papouin et al., 2012). These reported findings suggest that blocking D-serine signaling could downregulate NMDA receptor-dependent synaptic activation in CA3-CA1 areas and retrosplenial cortex, which are crucial for the memory retrieval and maintenance of remote fear memory. The way of changing extracellular D-serine levels has been used to modulate NMDA receptor-dependent synaptic activity (Meunier et al., 2017). In the mammalian brain, the synthesis of D-serine from L-serine is catalyzed by serine racemase (SRR) and SRR gene knockout (KO) mice exhibit a 90% reduction in the D-serine content in the hippocampus and cerebral cortex (Inoue et al., 2008). In one previous model of SRRKO mice, the magnitude of LTP at the medial perforant pathway to dentate gyrus (DG) synapses was diminished (Balu et al., 2013). Accordingly, we demonstrated that D-serine reduction in SRRKO mice attenuates a brain damage induced by NMDA receptor-mediated neurotoxicity (Inoue et al., 2008). Furthermore, we proved the role of D-serine in post-retrieval extinction of contextual fear memory (Inoue et al., 2018). Therefore, it is conceivable that the SRR deletion-induced D-serine reduction might downregulate the NMDA receptor signaling that is required for memory reconsolidation.

Adult hippocampal neurogenesis occurs in the subgranular zone of the DG in humans and every other mammalian species (Liu, 2022; Terreros-Roncal et al., 2022). Adult-born neurons in the DG functionally integrate into the hippocampal circuitry, playing a role in neuroplasticity (Ming and Song, 2005; Leuner and Gould, 2010). Growing evidence shows that adult-born hippocampal neurons are required for acquisition and expression of contextual fear memory (Saxe et al., 2006; Winocur et al., 2006; Drew et al., 2010). Advances in cell type-specific optogenetic or chemogenetic manipulation approaches have demonstrated that the newly generated immature granule cells in the DG contribute to the encoding or retrieval stages of DG-dependent contextual and spatial discrimination behaviors in an experience-dependent manner (Gu et al., 2012; Danielson et al., 2016; Anacker et al., 2018; Huckleberry et al., 2018). Noticeably, silencing during remote memory reconsolidation the population of adult-born neurons that were immature at the time of learning, impaired subsequent memory retrieval, suggesting a contribution of adult hippocampal neurogenesis in memory reconsolidation (Lods et al., 2021). These evidences suggest another possible approach to weaken remote fear memory by targeting retrieval-induced reconsolidation *via* the suppression of neurogenesis.

To the best of our knowledge, the studies exploring the role of either the NMDA receptor or hippocampal neurogenesis in the contextual fear memory, including fear memory acquisition and expression, as well as retrieval and extinction, are mostly in isolation, and no report has simultaneously investigated the involvement of the two factors in memory retrieval and remote fear memory (28-day-old memory) maintenance. In this study, aiming to weaken original fear memory, we used contextual fear conditioning with multiple retrievals intervention to induce reconsolidation, and targeted the induced reconsolidation by blocking the D-serine-mediated NMDA receptor signaling and hippocampal neurogenesis with different approaches. The present findings provide insights into the role of D-serine-mediated NMDA receptor signaling and neurogenesis in memory retrieval and remote fear memory maintenance, potentially contributing to the development of a new strategy to improve the efficacy of exposure-based therapy in PTSD treatment.

## Materials and methods

### Mice

We used male wild type (WT) and SRRKO mice at the age of 2 months with a pure C57BL/6N genetic background, as previously reported (Miya et al., 2008). D-Serine content in the cerebral cortex and hippocampus of SRRKO mice is approximately 10-fold lower than those in WT mice. By contrast, the levels of L-serine, glycine, and glutamate in the cerebral cortex and hippocampus of SRRKO mice are equivalent to those in WT mice (Inoue et al., 2008). Animal care and experimental protocols were approved by the Animal Experiment Committee of the University of Toyama (Authorization No. 2018 MED-38). The mice were kept in a temperature- and humidity-controlled room under a 12 h light/dark cycle (lights on at 7:00 a.m.) and had access to food and water *ad libitum*.

### Contextual fear conditioning, multiple retrievals, and remote fear memory test

Mice were handled daily for 1 min for a week before the behavioral experiments. Fear conditioning was conducted in a small conditioning chamber (10 × 10 × 10 cm; with transparent walls and a floor made of 14 stainless steel rods) surrounded by a sound-attenuating chest (CL-M3, O’Hara & Co., Ltd., Tokyo, Japan).

For the contextual fear conditioning, mice were placed in the conditioning chamber and two electrical footshocks (0.5 mA, 1 s) were delivered 59 and 119 s after the entry to the chamber as previously reported (Inoue et al., 2018). One minute after the last footshock, the mice were returned to their home cages. As a multiple retrieval group, the mice were re-exposed to the conditioning chamber for 5 min or 30 min without receiving a footshock 1, 7, and 14 days after fear conditioning, and testing remote fear memory was performed in the conditioned chamber for 5 min on day 28. As a non-retrieved group, the mice were fear-conditioned as described above, and the remote fear memory was tested in the conditioned chamber for 5 min 28 days after fear conditioning.

Mouse behavior was automatically recorded by a digital camera and the Freezeframe software (Coulbourn Instruments). Freezing responses were analyzed on a Macintosh computer with Image FZC 2.22sr2 (O’Hara & Co., Ltd.), the software based on the NIH Image program. For the experiments of fear conditioning, the brief retrievals with 5 min re-exposure, and the tests of remote fear memory, the capture rate of images was set 2 frames/sec. For the extended retrievals with 30 min re-exposure, the capture rate of images was set at 1 frame/sec.

### X-ray irradiation

X-ray irradiation was performed as previously reported (Santarelli et al., 2003), with modifications. Briefly, 8-week-old mice were anesthetized with 3.6% of chloral hydrate, placed in a stereotaxic frame, and exposed to cranial irradiation using an X-ray irradiation apparatus (MBR-1505R, HITACHI) operated at 150 kV and 5 mA. Mice were protected with a lead shield that covered the entire body except for a 3.2 × 11 mm treatment field above the hippocampus. The corrected dose rate was approximately 0.35 Gy/min at a source-to-skin distance of 13 cm. The procedure lasted for approximately 15 min, delivering a total of 5 Gy.

### Drug administration

Temozolomide (TMZ, LKT LAB, Toyama, Japan) was dissolved in dimethylsulfoxide (DMSO) and then diluted in saline (0.9% NaCl) to a concentration of 5 mg/ml. The dose of TMZ (25 mg/kg body weight) and the injection schedule were chosen based on the results of previous behavioral studies (Stone et al., 2011; Castilla-Ortega et al., 2016). TMZ or saline was administered intraperitoneally 24 h after fear conditioning for three consecutive days during the first and second weeks. Mice were weighed on every injection day and the TMZ or saline injection amounts were calculated.

1-Hydroxy-3-aminopyrrolodine-2-one (HA-966, RSD, Kyoto, Japan) was dissolved in saline at a concentration of 20 mg/ml, and was administered by the use of osmotic mini-pumps (model 1003D, Alzet, Cupertino, CA, USA) 14 days after contextual fear conditioning. For the osmotic pump implantation, mice were anesthetized with isoflurane (2–5%) and the osmotic pump was inserted subcutaneously at the back of the animals (the pump was parallel to the spine and the flow moderator directed posteriorly), and then the wound was sutured. Pumping rate was 0.25 μL/h and the pumping duration was 14 days. For control and TMZ groups, mice received sham operations.

### Immunofluorescent staining

The WT and SRRKO mice were deeply anesthetized with pentobarbital sodium (100 mg/kg body weight, intraperitoneal injection) and then transcardially perfused with phosphate-buffered saline (PBS, pH 7.4) followed by 4% paraformaldehyde (PFA) in 0.1 M phosphate buffer (PB, pH 7.4). The brains were removed, post-fixed with 4% PFA overnight, and dipped in 0.1 M PB containing 30% (w/v) sucrose for 36 h at 4°C. The brains were cut into 25-μm-thick serial coronal sections using a freezing microtome. For each animal, every eighth section (bregma from –1.34 to –3.58 mm, 12-sections/animal) was selected and prepared for the immunofluorescent staining as follows.

Free-floating brain sections were rinsed with PBS and blocked with Protein Block Serum Free (Dako Cytomation, Carpinteria, CA, USA) for 10 min at room temperature (RT). The brain sections were then incubated with a goat anti-doublecortin (DCX) polyclonal antibody (Santa Cruz Biotechnology, Santa Cruz, CA, 1:100) diluted in PBS containing 1% bovine serum albumin (BSA) overnight at 4°C. After washing in PBS, the sections were incubated with donkey anti-goat IgG conjugated with Alexa Fluor 488 (Invitrogen, Carlsbad, CA, USA, 1:500) diluted in PBS containing 1% BSA for 1 h at RT. The sections were then washed in PBS, counterstained with DRAQ5 (Abcam, Cambridge, Cambridgeshire, UK) for 30 min at RT. After washing in PBS, the sections were covered with a coverslip.

A confocal laser scanning microscope (Leica TCS-SP5, Leica Microsystems, Mannheim, Germany) was used to collect fluorescent image stacks. Three-dimensional image stacks (image size, 1.55 mm × 1.55 mm; pixel size, 3.03 μm × 3.03 μm, in each plane) were acquired in a step size of 2.5 μm. The DCX-positive cells were counted in a genotype-blind manner. The number of DCX-positive cells in the DG was calculated with the formula: DCX-positive cells/DG = DCX-positive cells/section × 96.

### Statistical analyses

Two-way repeated measures ANOVA was performed using Excel Statistics (Statcel 2; Social Survey Research Information Co. Ltd., Tokyo, Japan). Three-way repeated measures ANOVA was performed using the GraphPad Prism 9.0 software (GraphPad Software Inc., San Diego, CA, USA). Significant ANOVA results were followed by a *post hoc* Tukey–Kramer test for multiple comparisons. All values are presented as the mean ± SEM. Values of *p* < 0.05 were considered statistically significant (^*^p < 0.05, ^**^p < 0.01).

## Results

### Effect of simultaneous blockade of D-serine signaling and neurogenesis on remote fear memory following multiple retrievals

**FIGURE 1:**
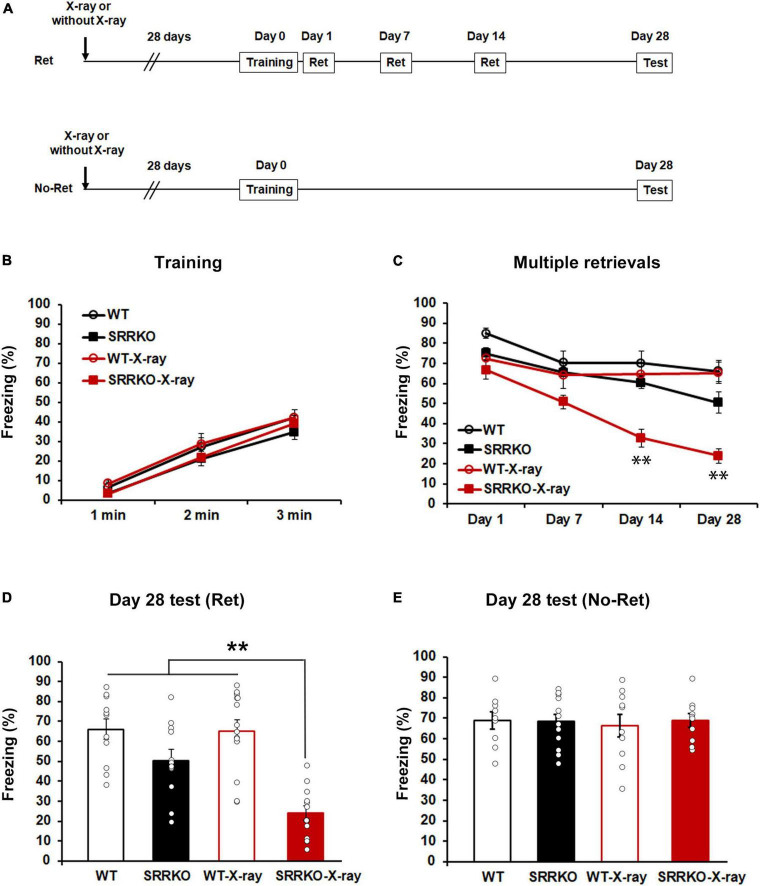
Hippocampal neurogenesis dampening attenuates remote fear memory following multiple retrievals in SRRKO mice. **(A)** Experimental schedule for X-ray irradiation, contextual fear conditioning, and multiple retrievals. Two different protocols, one with multiple retrievals (Ret), and another without memory retrieval (No–Ret), were used. **(B)** No significant difference was observed in freezing between WT (*n* = 11), SRRKO (*n* = 12), WT-X-ray (*n* = 14), and SRRKO-X-ray (*n* = 13) mice during fear conditioning. **(C)** During the course of four retrieval sessions, SRRKO-X-ray mice exhibited a significant reduction in freezing levels compared to other three groups in the tests on day 14 and 28. **(D)** During the remote test on day 28, SRRKO-X-ray mice exhibited significantly lower freezing levels compared to the other three groups. **(E)** Under the condition without intervention of multiple retrievals, no significant difference was observed in freezing level between four groups (WT, *n* = 9; SRRKO, *n* = 12; WT-X-ray, *n* = 10; SRRKO-X-ray, *n* = 10) during the remote memory test on day 28. Data are presented as the means ± SEM. ^**^p < 0.01.

We designed a paradigm to induce reconsolidation, with four sessions of 5 min retrieval conducted on days 1, 7, 14, and 28 after fear conditioning (Figure 1A). To investigate how D-serine reduction alone and combined with neurogenesis inhibition could affect retrieval-induced reconsolidation and eventually remote contextual fear memory, we conducted hippocampal X-ray irradiation to inhibit neurogenesis in WT and SRRKO mice 4 weeks before fear conditioning (Figure 1A). Mice were divided into four groups: WT mice receiving no X-ray irradiation (WT), SRRKO mice receiving no X-ray irradiation (SRRKO), WT mice receiving X-ray irradiation (WT-X-ray), and SRRKO mice receiving X-ray irradiation (SRRKO-X-ray). During the training, three-way repeated measures ANOVA revealed a significant effect of time [*F*_(2,92)_ = 165.1, *p* < 0.001] but not genotype [*F*_(1,46)_ = 3.394, *p* = 0.072], X-ray irradiation [*F*_(1,46)_ = 0.219, *p* = 0.642], time × genotype [*F*_(2,92)_ = 0.197, *p* = 0.822] and time × X-ray irradiation [*F*_(2,92)_ = 0.075, *p* = 0.928] interactions (Figure 1B). During the course of the four retrieval sessions, three-way repeated measures ANOVA revealed significant effects of genotype [*F*_(1,46)_ = 17.75, *p* < 0.001], retrieval sessions [*F*_(3,138)_ = 45.88, *p* < 0.001], X-ray irradiation [*F*_(1,46)_ = 10.49, *p* = 0.002], and retrieval sessions × genotype interaction [*F*_(3,138)_ = 11.16, *p* < 0.001] but not retrieval sessions × X-ray irradiation [*F*_(3,138)_ = 1.021, *p* = 0.386] and genotype × X-ray irradiation [*F*_(1,46)_ = 2.701, *p* = 0.107] interactions (Figure 1C). The *post hoc* analysis showed that SRRKO-X-ray mice exhibited a significant reduction in freezing levels compared to other three groups in the tests on days 14 and 28 (Figure 1C). During the remote test on day 28, two-way repeated measures ANOVA revealed a significant effects of genotype [*F*_(1,46)_ = 34.95, *p* < 0.001], X-ray irradiation [*F*_(1,46)_ = 7.835, *p* = 0.007], and genotype × X-ray irradiation interaction [*F*_(1,46)_ = 6.838, *p* = 0.012]. The *post hoc* analysis showed that SRRKO-X-ray mice exhibited a significant reduction in freezing levels compared to other three groups, and that in WT mice, the multiple retrievals alone or its combination with neurogenesis dampening resulted in no reduction in remote fear memory (Figure 1D). The non-retrieved mice (No-Ret) were used to examine how multiple retrievals (Ret) could affect remote fear memory maintenance. In the remote fear memory test on day 28, two-way repeated measures ANOVA revealed that there were no significant effects of genotype [*F*_(1,37)_ = 0.068, *p* = 0.796] and X-ray irradiation [*F*_(1,37)_ = 0.051, *p* = 0.823] (Figure 1E). These data suggest that simultaneous D-serine reduction and neurogenesis dampening resulted in a progressive decrease in freezing following each retrieval, reaching a significant attenuating effect on remote contextual fear memory on day 28.

**FIGURE 2:**
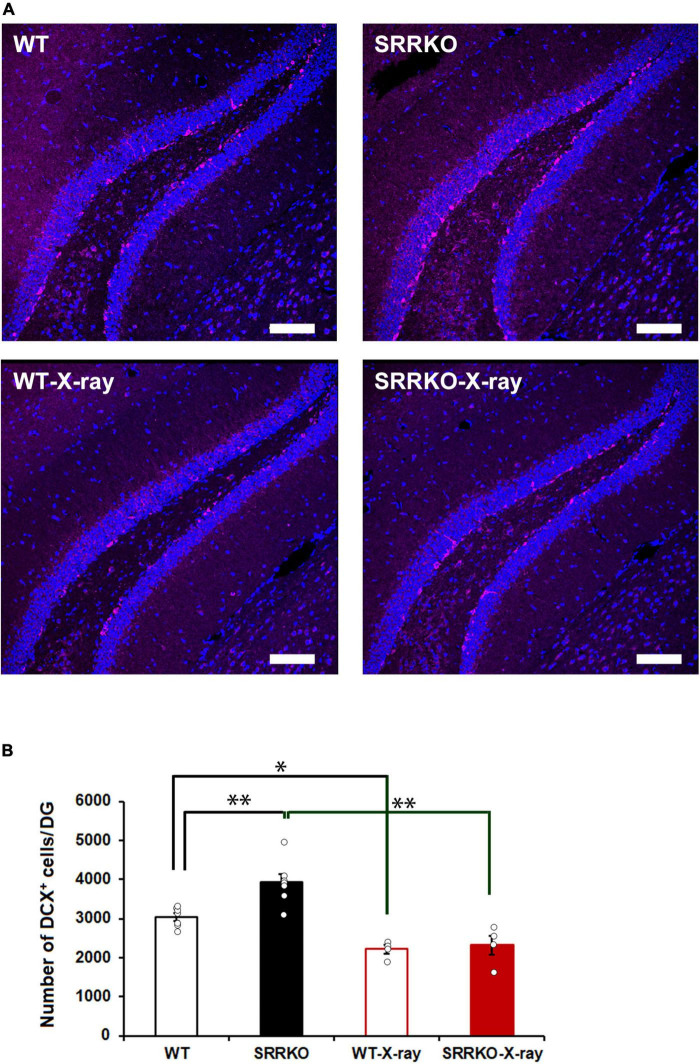
Effects of X-ray irradiation on adult hippocampal neurogenesis. **(A)** Immediately after the fourth retrieval on day 28, mouse brains of the four groups, WT, WT-X-ray, SRRKO, and SRRKO-X-ray, were removed and stained using anti-doublecortin (DCX) antibody (magenta) and DRAQ5 (blue). Scale bars, 100 μm. **(B)** Quantification of DCX-positive (DCX^+^) cells in the dentate gyrus (DG) of WT and SRRKO mice with or without X-ray irradiation (WT, *n* = 7; SRRKO, *n* = 7; WT-X-ray, *n* = 4; SRRKO-X-ray, *n* = 4). Data are presented as the means ± SEM. ^*^p < 0.05, ^**^p < 0.01.

To confirm X-ray irradiation efficiency, the brains were removed immediately after the completion of the final retrieval test on day 28 and subjected to DCX immunofluorescent staining. Two-way repeated measures ANOVA revealed significant effects of genotype [*F*_(1,18)_ = 11.176, *p* = 0.004], X-ray irradiation [*F*_(1,18)_ = 42.087, *p* < 0.001], and genotype × X-ray irradiation interaction [*F*_(1,18)_ = 4.530, *p* = 0.047]. The *post hoc* analysis showed that the X-ray irradiation significantly reduced the number of DCX-positive cells in both the WT (26.7 ± 3.7% reduction) and SRRKO (40.9 ± 6.2% reduction) mice (Figures 2A, B). Interestingly, we found a significant increase in the number of DCX-positive cells in SRRKO mice compared to WT mice (Figures 2A, B).

### Effect of pharmacological blockade of D-serine signaling and neurogenesis on remote fear memory following multiple retrievals

**FIGURE 3:**
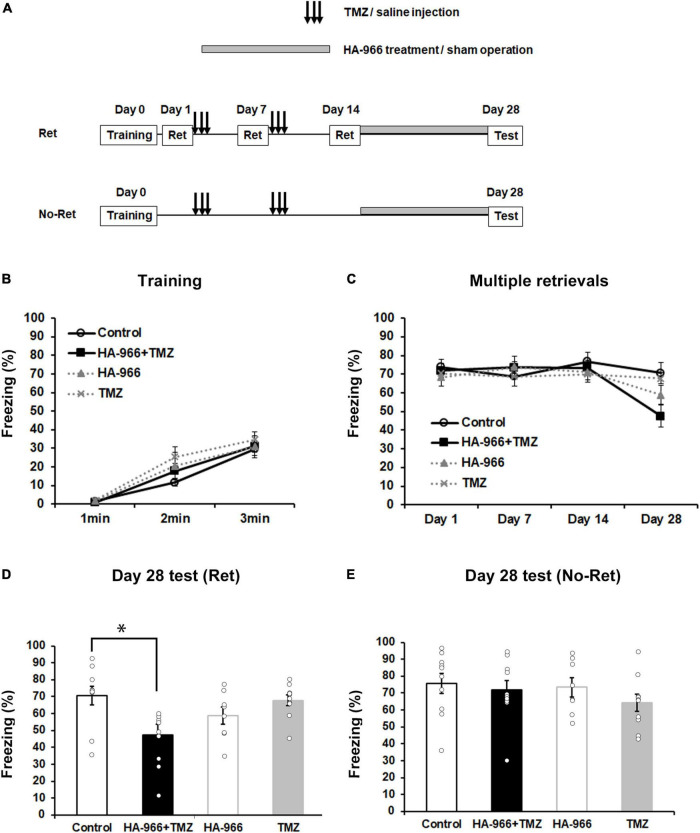
Pharmacological blockade of D-serine signaling and hippocampal neurogenesis attenuates remote fear memory following multiple retrievals. **(A)** Experimental schedule for contextual fear conditioning, multiple retrievals, and drug treatments. Two different protocols, one with multiple retrievals (Ret) and another without memory retrieval (No–Ret) were used. In the two behavioral tests, mice were divided into four groups: control (saline injection and sham operation), TMZ + HA-966 (TMZ injection and HA-966 administration), HA-966 (saline injection and HA-966 administration), and TMZ (TMZ injection and sham operation) group, respectively. **(B)** During the fear conditioning, no significant difference could be observed in the freezing level between the four groups (control, *n* = 10; HA-966 + TMZ, *n* = 10; HA-966, *n* = 8; TMZ, *n* = 10). **(C)** During the course of four retrieval sessions, mice treated with TMZ and HA-966 exhibited a significant decrease in freezing levels on day 28 than that on day 1. In contrast, multiple retrievals did not affect freezing level in the other three groups. **(D)** TMZ + HA966 group (*n* = 10) exhibited significantly lower freezing levels compared to the other three groups during memory test on day 28 following multiple retrievals. **(E)** Under the condition without intervention of multiple retrievals, no significant difference was observed in freezing level between four groups (control, *n* = 10; HA-966 + TMZ, *n* = 10; HA-966, *n* = 8; TMZ, *n* = 10) during the remote memory test on day 28. Data are presented as the means ± SEM. ^*^p < 0.05.

Based on the above-described data, we further attempted to use post-training pharmacological manipulation to block D-serine signaling and inhibit the neurogenesis so as to target the retrieval-induced reconsolidation, aiming to validate the possibility to translate the current approach into clinical applications. We used TMZ, an antimitotic drug to inhibit neurogenesis (Niibori et al., 2012; Egeland et al., 2017), and HA-966, an NMDA receptor antagonist to act on the glycine- and D-serine-binding site (Keith et al., 1989), to block D-serine signaling. TMZ injections were carried out for three consecutive days during the first and second weeks after fear conditioning (Figure 3A). The subcutaneous HA-966 administration using osmotic mini-pumps was started immediately after the completion of the retrieval test on day 14 and continued until the fourth retrieval test on day 28 (Figure 3A). According to different experimental treatments, mice were divided into four groups: control (saline injection and sham operation), combined (TMZ injection and HA-966 administration), HA-966 (saline injection and HA-966 administration), and TMZ (TMZ injection and sham operation) group. During fear conditioning, three-way repeated measures ANOVA revealed a significant effect of time [*F*_(2,68)_ = 93.86, *p* < 0.001] but not TMZ injection [*F*_(1,34)_ = 0.941, *p* = 0.339], HA-966 administration [*F*_(1,34)_ = 0.014, *p* = 0.905], time × TMZ injection [*F*_(2,68)_ = 0.803, *p* = 0.452], and time × HA-966 administration [*F*_(2,68)_ = 0.080, *p* = 0.923] interactions (Figure 3B). During the course of four retrieval sessions, three-way repeated measures ANOVA revealed significant effects of the retrieval sessions [*F*_(3,102)_ = 10.12, *p* < 0.001] and retrieval sessions × HA-966 administration interaction [*F*_(3,102)_ = 7.037, *p* < 0.001] but not TMZ injection [*F*_(1,34)_ = 0.411, *p* = 0.526], HA-966 administration [*F*_(1,34)_ = 0.941, *p* = 0.339], and retrieval sessions × TMZ injection interaction [*F*_(3,102)_ = 1.011, *p* = 0.391] (Figure 3C). The *post hoc* analysis showed that the combined group exhibited a significant reduction in freezing level in the fourth retrieval test on day 28 compared to those in the retrieval tests on day 1, 7, and 14. During the remote test on day 28, two-way repeated measures ANOVA revealed a significant effect of HA-966 administration [*F*_(1,34)_ = 10.578, *p* = 0.002] but not TMZ injection [*F*_(1,34)_ = 1.806, *p* = 0.188] and TMZ injection × HA-966 administration interaction [*F*_(1,34)_ = 0.687, *p* = 0.413] (Figure 3D). Under the condition without intervention of multiple retrievals (No-Ret), two-way repeated measures ANOVA revealed that there were no significant effects of HA-966 administration [*F*_(1,34)_ = 0.200, *p* = 0.657] and TMZ injection [*F*_(1,34)_ = 1.461, *p* = 0.235] in the remote fear memory test on day 28 (Figure 3E). These results validated the efficacy of the pharmacological manipulation-based targeting approach in weakening remote fear memory.

**FIGURE 4:**
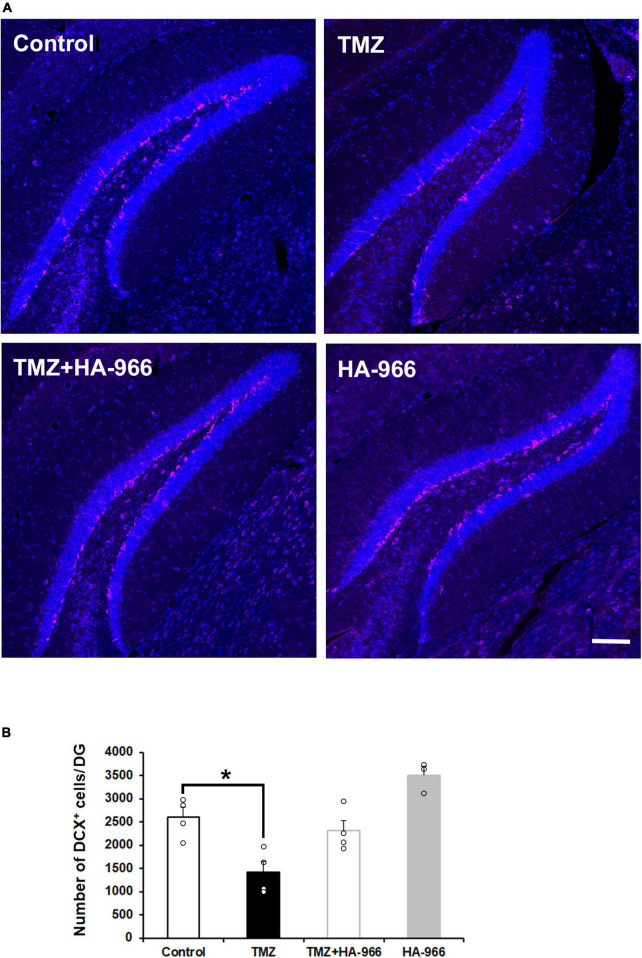
Effects of TMZ and HA-966 treatment on hippocampal neurogenesis. **(A)** Immediately after the fourth retrieval on day 28, mouse brains were removed and stained using anti-doublecortin (DCX) antibody (magenta) and DRAQ5 (blue). Scale bar, 100 μm. **(B)** Quantification of DCX-positive (DCX^+^) cells in the dentate gyrus (DG) of control (*n* = 4), TMZ (*n* = 4), TMZ + HA966 (*n* = 4), and HA-966 (*n* = 3) groups. Data are presented as the means ± SEM. *p < 0.05.

To confirm the TMZ injection efficacy on neurogenesis, the brains were removed immediately after the completion of the final retrieval test on day 28 and subjected to DCX immunofluorescent staining. Two-way repeated measures ANOVA revealed significant effects of HA-966 administration [*F*_(1,11)_ = 13.628, *p* = 0.004] and TMZ injection [*F*_(1,11)_ = 28.763, *p* < 0.001] but not TMZ injection × HA-966 administration interaction [*F*_(1,11)_ = 0.001, *p* = 0.977] (Figures 4A, B). The TMZ group showed approximately 45% reduction of DCX-positive cells in the DG compared to the control group.

### Effect of blockade of D-serine signaling and neurogenesis on remote fear memory following extended or brief retrievals

**FIGURE 5:**
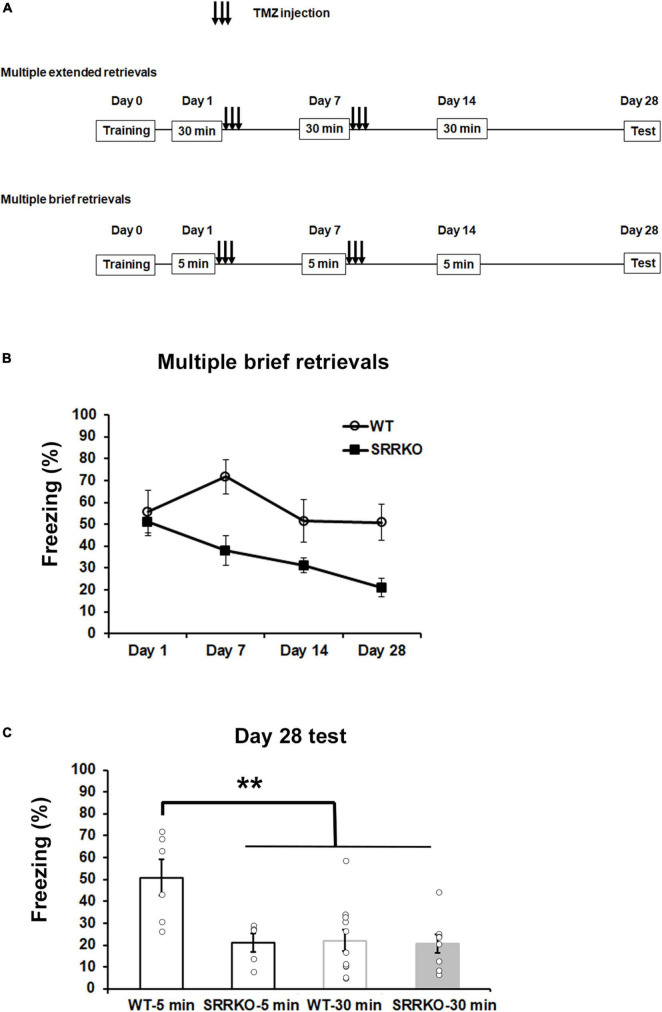
Effects of blockade of D-serine signaling and hippocampal neurogenesis on remote fear memory following extended and brief retrievals. **(A)** Experimental schedule for contextual fear conditioning, multiple retrievals, and TMZ treatment. Two different protocols, extended retrievals with 30 min re-exposure (as an extinction procedure) and brief retrievals with 5 min re-exposure, were used. **(B)** During the course of four brief retrievals on day 1, 7, 14, and 28, TMZ-treated SRRKO mice exhibited significantly lower freezing levels compared to TMZ-treated WT mice (WT, *n* = 6; SRRKO, *n* = 5). **(C)** During the remote test on day 28, WT mice receiving brief retrievals (WT-5 min, *n* = 6) exhibited a significantly higher freezing level compared to the other three groups, SRRKO mice receiving brief retrievals (SRRKO-5 min, *n* = 5), WT mice receiving extended retrievals (WT-30 min, *n* = 11), and SRRKO mice receiving extended retrievals (SRRKO-30min, *n* = 8). Data are presented as the means ± SEM. **p < 0.01.

Studies have demonstrated that in the model of contextual fear conditioning, memory retrieval initiates two opposing processes, reconsolidation and extinction, which are mainly dominated by duration of memory reactivation and strength of memory (Suzuki et al., 2004). Brief memory retrieval (e.g., 5 min re-exposure) induces reconsolidation, whereas extended retrieval (30 min re-exposure) causes an extinguishing effect. Therefore, we investigated if there is difference in effects of brief and extended retrievals on remote fear memory, under the conditions of blocking D-serine signaling and neurogenesis. We first examined the extinguishing effect of extended retrievals with 30 min re-exposure in WT and SRRKO mice, with injection of TMZ after fear conditioning (Figure 5A). During the course of three extinction procedures on day 1, 7, and 14, the two-way repeated measures ANOVA revealed a significant effect of extinction procedures [day 1, *F*_(5,102)_ = 9.90, *p* < 0.001; day 7, *F*_(5,102)_ = 4.90, *p* < 0.001; day 14, *F*_(5,102)_ = 3.67, *p* = 0.004] but not the genotype [day 1, *F*_(1,102)_ = 1.74, *p* = 0.190; day 7, *F*_(1,102)_ = 0.94, *p* = 0.334; day 14, *F*_(1,102)_ = 0.82, *p* = 0.368] and extinction procedures × genotype interaction [day 1, *F*_(5,102)_ = 0.67, *p* = 0.643; day 7, *F*_(5,102)_ = 0.70, *p* = 0.625; day 14, *F*_(5,102)_ = 0.71, *p* = 0.615] (Supplementary Figures 1A–C). For comparative analysis, we then used 5 min re-exposure for retrievals to repeat the same experiment. During the course of four retrieval sessions, the two-way repeated measures ANOVA revealed a significant effect of genotype [*F*_(1,36)_ = 16.08, *p* < 0.001] but not retrieval sessions [*F*_(3,36)_ = 2.72, *p* = 0.058] and genotype × retrieval sessions interaction [*F*_(3,36)_ = 1.38, *p* = 0.264] (Figure 5B). During the remote test on day 28, two-way repeated measures ANOVA revealed significant effects of retrieval time [*F*_(1,26)_ = 7.884, *p* = 0.009] and genotype × retrieval time interaction [*F*_(1,26)_ = 6.002, *p* = 0.021], and a trend for the effect of genotype [*F*_(1,26)_ = 4.11, *p* = 0.053]. The *post hoc* analysis showed that the WT mice receiving brief retrievals exhibited significantly higher freezing levels than other three groups, the SRRKO mice receiving brief retrievals, the WT mice receiving extended retrievals, and the SRRKO mice receiving extended retrievals (Figure 5C). Collectively, these data suggest that the brief retrievals presumably induce memory reconsolidation, which could be suppressed by simultaneous blockade of the D-serine signaling and neurogenesis.

## Discussion

In the model of contextual fear conditioning with the paradigm of multiple retrievals, we first examined the effects of blocking D-serine signaling and hippocampal neurogenesis dampening on remote contextual fear memory, respectively, by the genetic deletion of SRR and hippocampal irradiation before conditioning. We found two main results: (1) either D-serine reduction or neurogenesis dampening alone exerts no significant suppressive effect on remote fear memory, and (2) simultaneously conducting the two above-mentioned interventions can progressively reduce freezing following each retrieval, finally attenuating remote fear memory. Furthermore, in the same behavioral experiment, simultaneous pharmacological blockade of D-serine signaling and neurogenesis after conditioning yielded a similar result. These results suggest an efficient approach of weakening remote contextual fear memory by manipulating both D-serine signaling and neurogenesis to disrupt putatively induced reconsolidation following multiple retrievals.

To date, significant effort has been made to target the retrieving process aiming to induce extinction memory for the improvement of exposure-based therapeutic intervention in PTSD patients. The major limitation of memory extinction is that the original fear memory is not altered, only inhibited, and thus the maladaptive defensive behaviors could return following the passage of time (Vervliet et al., 2013; Morrison and Ressler, 2014). Therefore, reconsolidation blocking would be a potential alternative to directly weaken or disrupt original fear memory. In fact, in the SRRKO mice receiving no intervention of multiple retrievals (No-Ret group), focal X-ray irradiation failed to result in a significant impairment in remote contextual fear memory tested 28 days after conditioning. This result is presumably owing to that the way of reducing D-serine level is a moderate downregulation of NMDA receptor function and the irradiation reduced 26.7% and 40.9% of immature neurons in WT and SRRKO mice, respectively. Therefore, in the fear conditioning with a relatively high intensity (two 0.5 mA footshocks), the strategy of using intervention of multiple retrievals is required for weakening the remote fear memory. According to previously reported data showing that in the model of contextual fear memory, brief memory retrieval (e.g., by less than 5 min) induces reconsolidation (Suzuki et al., 2004), we selected 5 min re-exposure for each retrieval, and designed a paradigm with multiple retrievals conducted after consolidation of the initial fear memory, aiming to induce reconsolidation to be targeted by manipulating D-serine signaling and neurogenesis.

Advances in recent studies have demonstrated the key roles of NMDA receptor-mediated signaling in retrieving contextual fear memory shown by (1) being upstream in the signaling to regulate MAPK activation and in turn influence CREB phosphorylation (Kida et al., 2002; Wang and Peng, 2016) indispensable for memory reconsolidation, (2) GluN2B-containing NMDA receptor blocking prevents the destabilization of original contextual fear memory initiated by retrieval (Milton et al., 2013), and (3) blocking of GluN2A-containing NMDA receptors in the retrosplenial cortex prevents retrieving recent and remote contextual fear memory (Corcoran et al., 2011). Therefore, the strategy of targeting reconsolidation by downregulation of NMDA receptor signaling needs to at least avoid damaging GluN2B-containing NMDA receptors to render the process of destabilization/reconsolidation process successfully induced. D-Serine, as a co-agonist, was previously used to regulate NMDA receptor function (Inoue et al., 2008; Meunier et al., 2017) by changing its level in the extracellular environment. To downregulate NMDA receptor signaling, we used SRRKO mice with a 90% reduction in D-serine content in the hippocampus and cerebral cortex. We observed that D-serine reduction alone exerts no significant effect on remote fear memory attenuation following multiple retrievals, suggesting that other critical factors could potentially exist that play compensatory roles to supporting reconsolidation, e.g., hippocampal neurogenesis.

To further improve the strategy of targeting the reconsolidation process, we dampened neurogenesis by focal X-ray irradiation on the hippocampus 4 weeks before fear conditioning, which was previously shown to abolish 4–6-week-old newborn neurons and resulted in contextual fear memory impairment (Hernández-Rabaza et al., 2009; Denny et al., 2012). Previous data have shown that using irradiation to abolish or dampen adult neurogenesis may result in inflammatory reaction in dose-dependent manner (Mizumatsu et al., 2003; Hernández-Rabaza et al., 2009), and a single irradiation of 5 or 10 Gy on whole brain resulted in an increase of microglial activation 2 months after irradiation, but no production of new astrocytes or oligodendrocytes (Mizumatsu et al., 2003). Although our work did not assess the production of new astrocytes or oligodendrocytes, we selected a single irradiation of 5 Gy, which is considered as a clinically relevant dose (Mizumatsu et al., 2003) and is lower compared to those used in other studies (Hernández-Rabaza et al., 2009; Denny et al., 2012). The used focal irradiation on hippocampus is supposed to be able to avoid an irradiation-induced cognitive impairment. Supportive to our assumption, other works demonstrated that ablation of neurogenesis with focal irradiation and genetic method showed the similar behavioral effects (Saxe et al., 2006; Denny et al., 2012).

We assessed the effect of hippocampal irradiation alone and its combination with D-serine reduction on freezing following each retrieval and the remote fear memory tested on day 28. Interestingly, the irradiated SRRKO mice exhibited progressively decreased freezing following each retrieval and finally a great degree of freezing level reduction tested on day 28, but such effect was not observed in WT mice, suggesting that both D-serine signaling and hippocampal neurogenesis are required for reconsolidation process. This result leads to a question if the attenuating effect on fear memory results from extinction. To address this question, we used the extended retrievals with 30 min re-exposure, as an extinction procedure, to examine the difference in effects of brief and extended retrievals on remote fear memory, under the conditions blocking D-serine signaling and neurogenesis. We found that the extended retrievals could extinguish the fear memory under the conditions of blocking neurogenesis alone or together with D-serine signaling. In contrast, as shown in Figures 1D, 5C, the brief retrievals alone or its combination with neurogenesis dampening exerted no suppressive effect on remote fear memory in WT mice. These data suggest that the brief retrievals with 5 min re-exposure presumably induced memory reconsolidation, which could only be suppressed by simultaneous blockade of the D-serine signaling and neurogenesis. In consideration of the demerit of reducing D-serine level by general SRRKO, which involves the whole brain, further investigation is needed to selectively reduce D-serine level in the hippocampus and retrosplenial cortex in the same behavioral experiment, for example, by regional injection of adeno-associated virus expressing D-amino acid oxidase, so as to validate the region-specific role of D-serine -mediated NMDA receptor signaling in the maintenance of remote contextual fear memory.

Neurogenesis inhibition at different time points, before or after conditioning, reportedly exerts a differential effect on different hippocampus-dependent memory tasks (Arruda-Carvalho et al., 2011; Pan et al., 2012; Huckleberry et al., 2018), and the attempt to translate such a laboratory approach into the clinical application needs to establish a similar effective pharmacological intervention proper to be applied for patients. Therefore, we designed a second experiment to conduct post-training pharmacological manipulation to block D-serine signaling and neurogenesis to target reconsolidation and assessed its efficacy in reducing remote fear memory. We selected TMZ, an antimitotic drug used for the treatment of high-grade glioma in certain patients, to inhibit neurogenesis (Niibori et al., 2012; Egeland et al., 2017), and HA-966, an NMDA receptor antagonist acting on the glycine- and D-serine-binding site (Keith et al., 1989), to block D-serine signaling. We observed that remote fear memory tested during the day 28 test was significantly reduced in mice administered with both drugs but not in those that received a single administration of either of the drugs. Furthermore, TMZ injection resulted in approximately 45% reduction in the number of DCX-positive immature neurons in DG after the completion of remote memory tests, suggesting that dampened neurogenesis is probably associated with a putative impairment of retrieval-induced reconsolidation. Recently, Lods et al. (2021) proved that adult-born neurons immature during learning are necessary for spatial remote memory in rats, by demonstrating that specific silencing of the adult-born neurons immature during learning impairs remote memory that was retrieved 4 weeks after training and tested 2 days following the retrieval. This new finding supports our assumption that ablation or reduction of the adult-born neurons before or after conditioning may negatively modulate the process of retrieval-induced memory reconsolidation.

It should not be neglected that hippocampal irradiation alone exerted no significant impact on remote memory after multiple memory retrievals. We postulate that there would be compensatory effect deriving from the remaining immature neurons or even mature neurons, and under such conditions, intact NMDA receptor function would be necessary for supporting the memory process, probably due to its contribution to new spine formation of the newborn neurons and their heightened synaptic plasticity (Ge et al., 2007).

Different to the strategy of weakening or disrupting retrieval-induced reconsolidation by blockade of D-serine signaling and neurogenesis, recently, much attention has been paid to the facilitation of forgetting hippocampus-dependent memory by increase of adult hippocampal neurogenesis to enhance memory updating during retrieval (Akers et al., 2014; Ishikawa et al., 2016). Memantine is one of the antagonist of NMDA receptor, clinically used to treat Alzheimer’s disease, and has been shown to be an enhancer of the adult hippocampal neurogenesis and forgetting of contextual fear memory (Maekawa et al., 2009; Ishikawa et al., 2016). It is interesting that in the present work, a significant increase in the number of DCX-positive cells was observed in SRRKO mice compared to WT mice. However, such enhancement of neurogenesis did not result in significant reduction of freezing in the remote fear memory test on day 28. The inconsistence is presumably owing to the variations of increased levels of neurogenesis and intensity of fear conditioning, for example, a successful forgetting of contextual fear memory was achieved by a high dose of memantine in contextual fear conditioning with relatively low intensity (Ishikawa et al., 2016). These findings warrant a further study to compare the effects of the two conceptually opposite approaches on recent and remote contextual fear memory in animal model trained with same intensity.

In summary, we established a strategy to progressively weaken original contextual fear memory by targeting the memory reconsolidation process following each of the four spaced multiple retrievals, through simultaneous manipulation to downregulate D-serine-mediated NMDA receptor signaling and dampen the hippocampal neurogenesis. Our approach leads to a one-direction decrease in freezing over all retrieval sessions, showing a powerful effect to attenuate remote contextual fear memory. Since Pavlovian fear is an adaptive response to conditioned cue and context, the remote contextual fear memory tested in the present experiment should be considered as a memory process with normal neurobiology. Therefore, the efficiency of the current approach in weakening remote fear memory needs to be further validated in PTSD models, e.g., the contextual fear memory in the model of single prolonged stress (Yamamoto et al., 2008). Although HA-966 used in the present work is not appropriate for clinical applications, a recently reported SRR inhibitor of madecassoside, purified from medicinal plants could reportedly cross the blood-brain barrier in animal experiments and exhibits a neuroprotective effect (Rani et al., 2020). TMZ is an alkylating agent with excellent oral bioavailability, good penetration across the blood-brain barrier, and a low toxicity profile. Moreover, as a chemical agent, it was used for the treatment of high-grade glioma in certain patients without serious toxicity (Brada et al., 1999). In experimental animals, stroke-induced hippocampal neurogenesis was successfully inhibited by TMZ treatment (Cuartero et al., 2019). Our further study would aim to validate the efficacy of the two drugs (madecassoside and TMZ)-based approach in attenuating context-evoked fear memory.

## Data availability statement

The original contributions presented in this study are included in the article/Supplementary material, further inquiries can be directed to the corresponding author.

## Ethics statement

This animal study was reviewed and approved by Animal Experiment Committee of the University of Toyama.

## Author contributions

RI and XN performed the experiments and analyzed data. All authors designed the studies, wrote the manuscript, and approved the submitted version.

## Supplementary material

Supplementary Figure 1 Effects of blockade of D-serine signaling and hippocampal neurogenesis on fear memory during multiple extended retrievals. **(A–C)** During the course of three extinction procedures on day 1 **(A)**, 7 **(B)**, and 14 **(C)**, there was no significant difference in freezing levels between the two genotypes (WT, *n* = 11; SRRKO, *n* = 8). Data are presented as the means ± SEM.
