# Supplementary figures and images for: Blockade of D-serine signaling and adult hippocampal neurogenesis attenuates remote contextual fear memory following multiple memory retrievals in male mice
Source: Front Neurosci. 2023 Jan 4;16:1030702. doi: 10.3389/fnins.2022.1030702 (PMC9845639; doi:10.3389/fnins.2022.1030702)

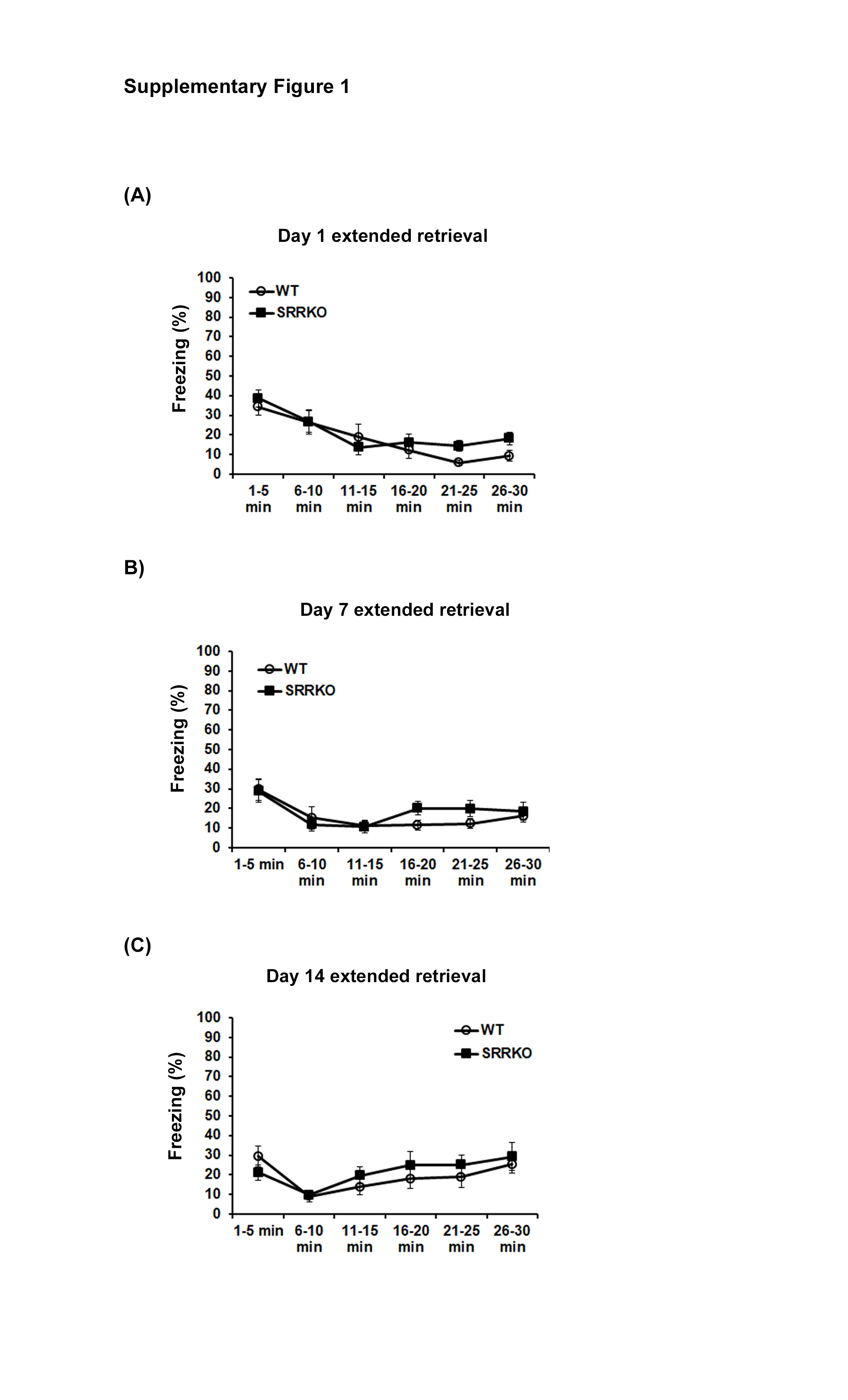

Supplement: Supplementary Figure 1 — Effects of blockade of D-serine signaling and hippocampal neurogenesis on fear memory during multiple extended retrievals. (A–C) During the course of three extinction procedures on day 1 (A), 7 (B), and 14 (C), there was no significant difference in freezing levels between the two genotypes (WT, n = 11; SRRKO, n = 8). Data are presented as the means ± SEM. [file Image_1.TIF]
